# Role of ferroptosis in pregnancy related diseases and its therapeutic potential

**DOI:** 10.3389/fcell.2023.1083838

**Published:** 2023-03-08

**Authors:** Jinfeng Xu, Fan Zhou, Xiaodong Wang, Chunheng Mo

**Affiliations:** ^1^ Department of Obstetrics and Gynecology, West China Second University Hospital, Sichuan University, Chengdu, China; ^2^ West China School of Medicine, Sichuan University, Chengdu, China; ^3^ Key Laboratory of Birth Defects and Related Diseases of Women and Children (Sichuan University), Ministry of Education, West China Second University Hospital, Sichuan University, Chengdu, China

**Keywords:** ferroptosis, molecular mechanisms, pregnancy related diseases, intrahepatic cholestasis of pregnancy (ICP), therapeutic potential

## Abstract

Ferroptosis is a form of regulated cell death characterized by iron overload, overwhelming lipid peroxidation, and disruption of antioxidant systems. Emerging evidence suggests that ferroptosis is associated with pregnancy related diseases, such as spontaneous abortion, pre-eclampsia, gestational diabetes mellitus, intrahepatic cholestasis of pregnancy, and spontaneous preterm birth. According to these findings, inhibiting ferroptosis might be a potential option to treat pregnancy related diseases. This review summarizes the mechanisms and advances of ferroptosis, the pathogenic role of ferroptosis in pregnancy related diseases and the potential medicines for its treatment.

## 1 Introduction

**FIGURE 1:**
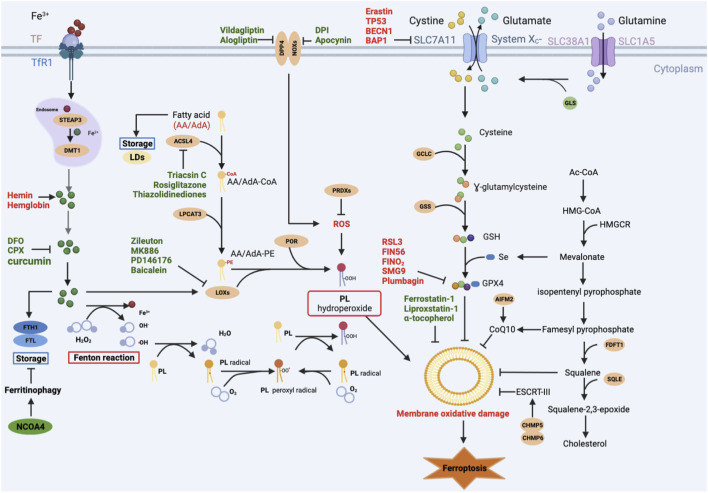
Main signaling pathways of ferroptosis. Ferroptosis can occur through three major pathways: 1) Iron overload: Fe^2+^ may directly generate excessive lipid reactive oxygen species (ROS) through the Fenton reaction, or Fe^2+^ acts as a cofactor of lipoxygenase (LOX) or prolyl hydroxylase, leading to lipid peroxidation and oxygen homeostasis. 2) Lipid peroxidation: ACSL4 catalyzes the ligation of CoA into free AA/AdA to form AA/AdA-CoA. AA/AdA-CoA are esterified into PE by LPCAT3 to form AA/AdA-PE. PE-AA/AdA-OOH are produced by the peroxidation of the AA/AdA-PE through non-enzymatically autoxidation (Fenton reaction) or enzyme-mediated pathways (LOXs). 3) Antioxidant systems: the SLC7A11-GSH-GPX4 axis, CoQ10 system, and other antioxidants like AIFM2, squalene. Red-colored: ferroptosis inducers; Green-colored: ferroptosis inhibitors.

**FIGURE 2:**
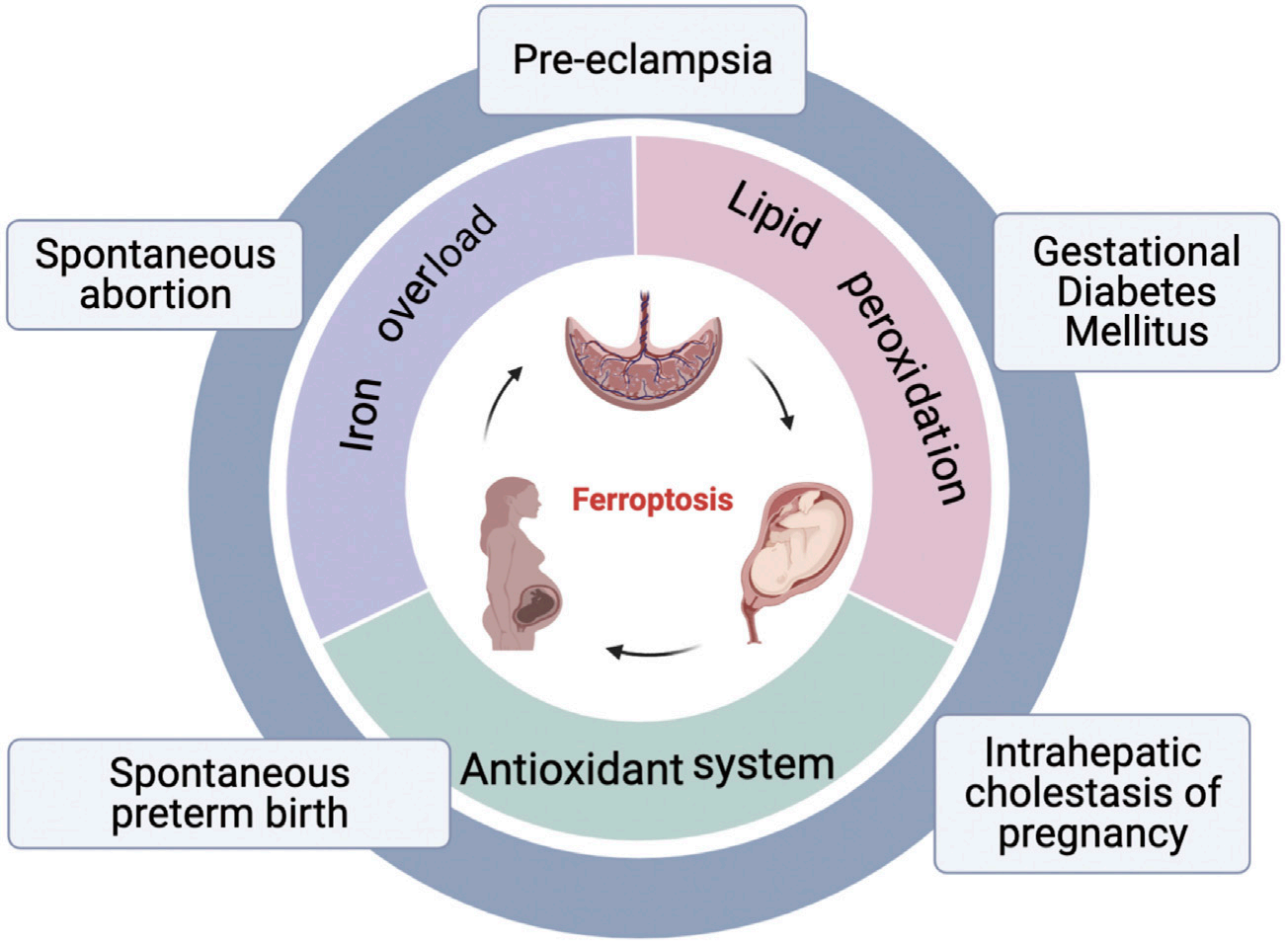
An overview of ferroptosis and pregnancy related diseases. Three hallmarks of ferroptosis: lipid peroxidation, iron overload, and disorder of antioxidant systems; Pregnancy related diseases associated with ferroptosis: spontaneous abortion, pre-eclampsia, gestational diabetes mellitus, intrahepatic cholestasis of pregnancy, spontaneous preterm birth.

Cells are the fundamental organizing unit of life. Cell death, is therefore of critical importance in diverse aspects of mammalian development and homeostasis. Ferroptosis is a form of regulated cell death, coined in 2012. Generally, it is mainly characterized by iron overload, overwhelming lipid peroxidation, and disruption of antioxidant systems, particularly depletion of glutathione peroxidase 4 (GPX4) (Long et al., 2022). Mounting evidence suggests that ferroptosis plays an important role in cancer (Lei et al., 2022), neurodegenerative diseases (Ryan et al., 2022), and ischemia/reperfusion injury, such as acute kidney injury (Fan et al., 2022), acute myocardial infarction (Miyamoto et al., 2022), and hepatic ischemia-reperfusion injury (Ye et al., 2022a), and autoimmune disease, like psoriasis (Zhou et al., 2022) and rheumatoid arthritis (Long et al., 2022), Serving as the maternal-fetal interface, placenta plays a central role in maternal and fetal health during pregnancy. Placenta insufficiency, however, is tightly associated with pregnancy related diseases (PRDs), such as pre-eclampsia, gestational diabetes mellitus (GDM), and intrahepatic cholestasis of pregnancy (ICP) (Cindrova-Davies and Sferruzzi-Perri, 2022). Recently, there has been a growing appreciation for the importance of ferroptosis in PRDs. In this review, we summarized the molecular mechanisms of ferroptosis, as well as its pathogenic role and potential medicines in PRDs (Figure 1; Figure 2).

## 2 Molecular mechanisms of ferroptosis

### 2.1 Lipid peroxidation

Mammalian lipid bilayers consist of up to 62% of unsaturated fatty acids of which 35% are polyunsaturated fatty acids (PUFAs) (Hulbert et al., 2002). However, PUFA is a double-edged sword. On the one hand, PUFAs are necessary for cell membrane to maintain its fluidity (Gill and Valivety, 1997) or deposit in lipid droplets (LDs) in order to produce metabolic energy in case of insufficient energy sources (Thiele and Spandl, 2008). On the other hand, PUFAs, especially arachidonic acid (AA) and adrenic acid (AdA), promote lipid peroxidation under various pathophysiological contexts (Doll et al., 2017). Acyl-CoA synthetase long chain family member 4 (ACSL4) (Küch et al., 2014) and lysophosphatidylcholine acyltransferase 3 (LPCAT3) (Hishikawa et al., 2008) are tightly linked to lipid peroxidation of AA/AdA. Firstly, ACSL4 catalyzes the ligation of CoA into free AA/AdA to form AA/AdA-CoA derivatives (Kagan et al., 2017). Then, AA/AdA-CoA are esterified into phosphatidylethanolamine (PE) by LPCAT3 to form arachidonic acid-phosphatidylethanolamines (AA/AdA-PE) (Hishikawa et al., 2008). Finally, toxic phospholipid hydroperoxides (PE-AA/AdA-OOH) are produced by the peroxidation of the AA/AdA-PE through non-enzymatically autoxidation or enzyme-mediated pathways (Dixon et al., 2012; Yang et al., 2016).

The non-enzymatic phospholipid (PL) autoxidation is iron-dependent lipid peroxidation. Hydroxyl radicals, produced by the interaction of Fe^2+^ and H_2_O_2_ (Fenton reaction), subtract hydrogen from lipid to form lipid radicals (L•) (Diggle, 2002). After that, the lipid radical combine with O_2_ to form a lipid peroxyl radical (LOO•), which then interacts with adjacent PUFAs to form lipid hydroperoxide (LOOH), and many electrophilic species such as malondialdehyde (MDA), and 4-hydroxynonenal (4HNE) (Rice-Evans and Burdon, 1993; Michalski et al., 2008).

Lipid peroxidation also occurs in enzyme-mediated processes. Lipoxygenases (LOXs), a dioxygenase containing non-heme iron, has six isoforms in humans: 15-LOX-1, 15-LOX-2, 12-LOX-1, 12-LOX-2, E3-LOX, and 5-LOX (Funk et al., 2002). Although the key role of LOXs in ferroptosis is still controversial, certain LOXs can catalyze the stereospecific addition of oxygen onto PUFAs (Kuhn et al., 2005), indicating LOXs may mediate ferroptosis. Indeed, LOX15, binding to the partner phosphatidylethanolamine-binding protein 1 (PEBP1), is of importance for erastin- or RSL3-induced ferroptosis (Wenzel et al., 2017). Furthermore, ferroptosis can be inhibited by some LOXs inhibitors, such as Zileuton, MK886, PD146176, Baicalein (Battista et al., 2012; Shah et al., 2018). However, some reported *in vivo* model of acute renal failure, 12/15- LOX deletion cannot eliminate the cell death of GPX4 knockout mouse (Friedmann Angeli et al., 2014; Brütsch et al., 2015). Therefore, the role of LOXs in ferroptosis should be further investigated. Other oxygenases, such as NADPH oxidases (NOXs) and cytochrome P450 oxidoreductase (POR), may also lead to ferroptosis. Apocynin and diphenyleneiodonium (DPI), two NOXs inhibitors, can directly mitigate ferroptotic cell death (Hou et al., 2019). Similarly, alogliptin and vildagliptin indirectly suppress NOXs activity mediated by dipeptidyl peptidase-4 (DPP-4), reducing lipid peroxidation (Zhang et al., 2022a). POR, identified through CRISPR/Cas9-mediated suppressor screening, can play a role in erastin-, FIN56-, ML210-, or Ras selective lethal small molecule 3 (RSL3)-induced ferroptosis, as well (Zou et al., 2020).

### 2.2 Iron in ferroptosis

Iron overload is a hallmark of ferroptosis. Iron drives ferroptosis mainly by two ways. Iron may directly generate excessive lipid reactive oxygen species (ROS) through the Fenton reaction (Conrad and Pratt, 2019). What’s more, Fe^2+^ acts as a cofactor of LOXs or prolyl hydroxylase (Doll and Conrad, 2017), which are enzymes responsible for lipid peroxidation and oxygen homeostasis (Kagan et al., 2017). Consequently, Fe^2+^ promotes the production of lipid ROS and contributes to ferroptosis indirectly. Therefore, iron metabolism, including iron uptake, transportation, utilization, may affect cell susceptibility to ferroptosis. Firstly, Fe^3+^ imports by binding to transferrin (TF), which can be recognized by transferrin receptor-1 (TfR1) in the cell membrane. And then, Fe^3+^ endocytosis in endosomes, where it is reduced to Fe^2+^ by six-transmembrane epithelial antigens of the prostate 3 (STEAP3). Finally, Fe^2+^ is transported to the cytosolic labile iron pool *via* divalent metal transporter 1 (DMT1) (El Hout et al., 2018). Fe^2+^ also comes from hemin and hemoglobin *via* the lysis of red blood cells, leading to ferroptosis (Kwon et al., 2015). It can be used in cellular processes or stored into ferritin, consisting of ferritin light chain (FTL) and ferritin heavy chain 1 (FTH1) (Ryu et al., 2017). But ferritin can be degraded by lysosomes through nuclear receptor coactivator 4 (NCOA4)-mediated ferritinophagy (Hou et al., 2016). Ferroportin (FPN1) is responsible for exporting iron (Donovan et al., 2000), resisting to ferroptosis.

### 2.3 Antioxidant systems

#### 2.3.1 The SLC7A11-GSH-GPX4 axis

The SLC7A11-GSH-GPX4 axis is a classical signaling pathway of ferroptosis (Dixon et al., 2012). Glutathione peroxidase 4 (GPX4), a glutathione (GSH) -dependent selenoenzyme, functions as a phospholipid hydroperoxidase to reduce toxic PE-AA/AdA-OOH to the corresponding non-toxic phospholipid alcohol (PLOH), inhibiting ferroptosis (Ursini et al., 1985). However, the antioxidant activity of GPX4 demands the catalytic selenocysteine (Sec) residue at 46 (U46) and two electrons supplied mainly by GSH (Maiorino et al., 2018). Sec, encoded by the UGA codon, is a major form of selenium (Se) in the cell. Generally, it is present at active sites of enzymes, catalyzing redox reactions, thereby eliminating hydroperoxides (Santesmasses et al., 2020). In addition, Se can upregulate the expression of GPX4 through transcription factor AP-2 gamma (TFAP2C) and specificity protein 1 (SP1) (Alim et al., 2019). Truly, translation of GPX4 is attenuated in LRP8KO cells due to the limiting Se (Li et al., 2022a). GSH, as the reducing agent, is the substrate for the lipid repair function of GPX4, lowering the risk of ferroptosis. The biosynthesis of GSH is based on glutamate, glycine and cysteine, which is tightly correlated with its precursor cystine and system Xc− (Lu, 2013). The Xc− system is an antiporter on the cell membrane composed of SLC7A11 and SLC3A2, transporting glutamate outwards and cystine inwards at 1:1 ratio (Bannai, 1986). Thus, factors, directly or indirectly inhibiting GPX4, play a key role in inducing ferroptosis. Erastin inhibits its activity by binding to SLC7A11, reducing cystine import, thereby reducing GSH synthesis (Yang et al., 2014). Tumor suppressor genes TP53, BECN1, BAP1 downregulate the expression of SLC7A11 to induce ferroptosis (Jiang et al., 2015; Song et al., 2018; Zhang et al., 2018). However, the nuclear transcription factor 2 (Nrf2) upregulates the expression of SLC7A11 to inhibit ferroptosis (Chen et al., 2017). High calcium and phosphate can downregulate the expression of GPX4, inducing ferroptosis (Ye et al., 2022b). RSL3 can covalently bind to GPX4, resulting in increasing lipid peroxidation (Yang et al., 2014). GPX4 also can be degraded by some compounds, such as FIN56, FINO2, plumbagin, SMG9 (Gaschler et al., 2018; Han et al., 2021; Sun et al., 2021; Zhan et al., 2022). Together, the SLC7A11-GSH-GPX4 axis is of great significance for ferroptosis.

#### 2.3.2 The FSP1 pathway

The ferroptosis suppressor protein 1 (FSP1)–NAD(P)H–coenzyme Q10 (CoQ10) pathway, acting in parallel to the SLC7A11-GSH-GPX4 pathway, is a potent suppressor of lipid peroxidation and ferroptosis (Bersuker et al., 2019; Doll et al., 2019). FSP1, known as apoptosis-inducing factor mitochondrial 2 (AIFM2), plays a crucial role in the non-mitochondrial CoQ antioxidant system (Wu et al., 2002). As members of the AIF family, FSP1 contains a short N-terminal hydrophobic sequence and a canonical flavin adenine dinucleotide (FAD)-dependent oxidoreductase domain, possessing NADH: ubiquinone oxidoreductase activity (Elguindy and Nakamaru-Ogiso, 2015). Ubiquinol, the reduced form of ubiquinone, known as CoQ10 traps lipid peroxyl radicals, thereby mediating lipid peroxidation. However, FSP1 catalyzes the catalyzing NADH: ubiquinone oxidoreductase reactions, reducing ubiquinol to CoQ10, which is a good radical-trapping antioxidant for lipid peroxides (Bersuker et al., 2019; Doll et al., 2019). Indeed, some reported that FSP1 inhibits ferroptosis in across hundreds of cancer cell lines and in mouse tumor models (Bersuker et al., 2019).

## 3 Ferroptosis inhibitors

Ferroptosis is associated with a great number of diseases. Multiple genes and signaling pathways, associated with lipid and iron metabolism, and antioxidant systems, play a role in inhibiting ferroptosis, providing new potential therapeutic drugs for these diseases (Table 1).

**TABLE 1:** Summary of ferroptosis inhibitors

Compounds/drugs	Model	Mechanism	Potential for PRDs	Reference
Lipid peroxidation				
ferrostatin-1, liproxstatin-1, SRS16-86	cell line: HT1080; HK-2; primary human renal proximal tubule epithelial cells;	inhibit lipid peroxidation	PE	Dixon et al. (2012), Friedmann Angeli et al. (2014)
deuterated PUFA	APP/PS1 mice	inhibit lipid peroxidation	NR	Raefsky et al. (2018)
thiazolidinediones	TAM-inducible Gpx4 ^−/−^ cells; TAM-inducible Gpx4^−/−^ mice	decrease the level of AA-CoA/AdA-CoA	GDM	Doll et al. (2017)
zileuton	HT22 cells	inhibit 5-LOX	NR	Long et al. (2022)
vitamin E,α-Tocopherol	SD rats with PTZ-Induced Epilepsy; Gpx4^flox/flox^C57BL/6 mice;	inhibit 15-LOX	PE	Hu et al. (2021a), Zhang et al. (2022b)
baicalein	HT22 cells, TBI mice model	inhibit 12/15-LOX	NR	Probst et al. (2017), Kenny et al. (2019)
PD-146176	human spermatozoa	inhibit 15-LOX	NR	Walters et al. (2018)
**Iron**				
deferiprone, deferoxamine, ciclopirox	HT-1080	reduce intracellular iron	PE	Long et al. (2022)
eriodictyol	APPswe/PS1E9 transgenic mice; HT-22 hippocampal cells	reduced intracellular iron accumulation	NR	Li et al. (2022b)
**Antioxidant systems**				
β-mercaptoethanol	OT-1 CD8þ T cell	drive a highly efficient cystine/cysteine redox cycle.	NR	Sha et al. (2015)
selenium	BTBR mouse model of ASD	enhance the number of selenoproteins	ICP	Wu et al. (2022)
cycloheximide	B35 neuroblastoma cells; 9L gliosarcoma cells	increased levels of GSH	NR	Rashad et al. (2022)
XJB-5–131	C57BL/6 mice of Renal I/R model	increase the expressions of GPX4	NR	Zhao et al. (2020)
1,25(OH)_2_D_3_	zebrafish liver cell line	increase the expressions of GPX4	SA, GDM, PE	Cheng et al. (2021)
astaxanthin	primary chondrocytes; SD rat model of osteoarthritis	increase the expressions of GPX4	PE	Wang et al. (2022)
echinatin	primary rat hippocampal neurons; SD rats	increase the expressions of GPX4	NR	Xu et al. (2022)
quercetin	KA-induced seizures in C57BL/6J mice; cell line: HT22	increase the expressions of GPX4	PE	Xie et al. (2022)
**CoQ10, idebenone**	NCI-H460; HT1080 cells; MDCK cells	inhibit lipid peroxidation	ICP	Bersuker et al. (2019), Doll et al. (2019), Schreiber et al. (2019)
**Membrane repair**				
vildagliptin	intracerebral hemorrhage C57BL/6 mice	inhibit DPP4	NR	Zhang et al. (2022a)
**Other antioxidants**				
BAPTA-AM	cell line:HK-2 cells; TCE-sensitization BALB/c mice	inhibit lipid peroxidation	NR	Liu et al. (2022)

PE, pre-eclampsia: gestational hypertension with proteinuria > 0.3g/L/day in the absence of a urinary tract infection or the abrupt onset of hypertension and proteinuria after 20 weeks of gestation (ACOG Practice Bulletin, 2019).

GDM, gestational diabetes mellitus: diabetes first diagnosed in the second or third trimester of pregnancy that is not clearly either preexisting type 1 or type 2 diabetes (American Diabetes Association, 2018).

ICP, intrahepatic cholestasis of pregnancy: characterized by maternal pruritus and increased serum bile acid concentrations, typically resolving postpartum (Chappell et al., 2019).

SA, spontaneous abortion: pregnancy loss at less than 20 weeks’ gestation in the absence of elective medical or surgical measures to terminate the pregnancy (Wilcox et al., 1988).

abrAbbreviations: AA, arachidonic acid; AdA, adrenic acid; PRDs, pregnancy related diseases; GPX4, Glutathione peroxidase 4; GSH, glutathione; LOX, lipoxygenase; PUFA, polyunsaturated fatty acid; SD, sprague-dawley.

### 3.1 Inhibition of lipid peroxidation

PUFAs, substrates of lipid peroxidation, are responsible for ferroptosis. Selectively bis-allylic deuterated PUFA, suppressing ferroptosis induced by RSL3 and erastin (Yang et al., 2016), is a promising therapeutic strategy against ferroptosis. Additionally, blocking the process of PUFAs incorporation into phospholipid membranes can reduce lipid peroxidation, like thiazolidinediones, rosiglitazone and Triacsin C (Van Horn et al., 2005; Angeli et al., 2017; Doll et al., 2017), which inhibits ACSL4. There are many LOXs inhibitors, such as vitamin E, α-Tocopherol, baicalein, PD-146176 (Probst et al., 2017; Walters et al., 2018; Kenny et al., 2019; Hu et al., 2021a; Zhang et al., 2022b), stopping the process of LOX-mediated lipid peroxidation, thereby resisting ferroptosis. Ferrostatins and liproxstatins are classical ferroptosis inhibitor, depressing lipid peroxidation (Zilka et al., 2017).

### 3.2 Iron chelator

Fe^2+^ overload in intracellular iron pools, like endoplasmic reticulum (ER), may trigger ferroptosis (Tang et al., 2021). However, intracellular iron accumulation is linked with the transport of extracellular iron. Recently, Li et al. found eriodictyol can significantly decrease TfR1 and FTH, and increase FPN, leading to resisting ferroptosis (Li et al., 2022b). Furthermore, iron chelators, such as deferoxamine (DFO), ciclopirox (CPX), and curcumin (Rainey et al., 2019) can reduce the concentration of Fe^2+^ (Yang and Stockwell, 2008), suppressing ferroptosis.

### 3.3 Antioxidant systems

Antioxidant systems protecting cell from oxidative damage in ferroptosis are associated with multiple enzymes and proteins, including SLC7A11-GSH-GPX4 axis, CoQ10 system (Kuang et al., 2020).

#### 3.3.1 The SLC7A11-GSH-GPX4 axis

The SLC7A11-GSH-GPX4 axis is the main ferroptosis prevention system. The induction of GPX4 synthesis is a classic pathway to suppress ferroptosis, relating to cysteine, system Xc-, Se, GSH. The β-mercaptoethanol may play a role in inhibiting ferroptosis by driving a highly efficient cystine/cysteine redox cycle (Sha et al., 2015). Cycloheximide is a potent inhibitor of ferroptosis, by increasing the concentration of GSH (Rashad et al., 2022). The XJB-5-131 alleviates I/R-induced renal injury and inflammation in mice by increasing the expression of GPX4 (Zhao et al., 2020). Similarly, 1,25(OH)_2_D_3_, astaxanthin, and echinatin can become resistant to ferroptosis by increasing GPX4 level (Cheng et al., 2021; Wang et al., 2022; Xu et al., 2022). At the same time, the 5-(tetradecyloxy)-2-furoic Acid (TOFA) was also found to be a potent suppressor of ferroptosis, through inhibiting the loss of GPX4 (Shimada et al., 2016). Quercetin, a natural polyphenol, could attenuate seizure-induced neuron ferroptosis *in vivo* and *in vitro* at *via* the SIRT1/Nrf2/SLC7A11/GPX4 axis (Xie et al., 2022).

#### 3.3.2 CoQ10 System

The FSP1–NAD(P)H–CoQ10 pathway, acting in parallel to the GPX4 axis, is a powerful antioxidant system in membrane structures (Teran et al., 2018). Idebenone, an analog of CoQ10, prevents ferroptosis caused by FIN56 or RSL3 (Shimada et al., 2016). Likewise, farnesyl pyrophosphate, an upstream product of CoQ10 synthesis, suppresses FIN56-induced ferroptosis (Shimada et al., 2016).

#### 3.3.3 Other antioxidants

AIFM2, promoting CHMP5- and CHMP6-mediated ESCRT-III membrane repair, results in blocking ferroptosis (Dai et al., 2020). As reported, some antioxidant proteins may also resist to ferroptotic cell death, like peroxiredoxins (Qi et al., 2019; Lovatt et al., 2020), thioredoxin (Llabani et al., 2019). Cytosolic Ca^2+^ overload was a key mediator of ferroptosis (Chen et al., 2020; Liu et al., 2022). Recently, researchers found BAPTA-AM, an intracellular Ca^2+^ chelator, could rescued ferroptosis in HK-2 cells (Liu et al., 2022). Therefore, limiting oxidative damage of ferroptosis has been a promising therapeutic strategy for PRDs.

## 4 Role of ferroptosis in pregnancy related diseases

Recently, basic research on ferroptosis in PRDs has gradually increased. Studies have indicated that placenta is susceptible to ferroptosis. Primarily, lipid peroxidation is frequent in placental injury (Schoots et al., 2018); Secondly, trophoblasts are abundant of iron: syncytiotrophoblasts extraordinarily highly expressed TfR1 (Seligman et al., 1979). Furthermore, Zrt- and Irt-like protein 8 (ZIP8) and Zrt- and Irt-like protein 14 (ZIP14), both of which play a roles in exporting iron from placental endosomal into the cytosol, are found at high levels in human placenta (Jenkitkasemwong et al., 2012). Three reviews described details on the role of iron and ferroptosis in the placenta (Ng et al., 2019; Beharier et al., 2021; Zaugg et al., 2022). Finally, decreased GPX4 levels have been associated with human placental dysfunction (Zhang et al., 2020). Therefore, fully understanding the role of ferroptosis in placenta dysfunction may provide new treatment options for PRDs, including spontaneous abortion, PE, GDM, ICP, and spontaneous preterm birth (Figure 2).

### 4.1 Pre-eclampsia

#### 4.1.1 Lipid peroxidation and pre-eclampsia

PE plays a leading role in maternal morbidity and mortality (Leitao et al., 2022). Ferroptosis has been related to the pathogenesis of PE (Chen et al., 2022). There are mounting evidence suggesting lipid peroxidation, is a major contributor for the damage of PE. A single-cell transcriptomics of the human placenta analysis indicated LPCAT3 and Sat1 (spermidine/spermine N1-acetyltransferase 1) highly expressed in trophoblasts (Ng et al., 2019), both of which are related to ferroptosis (Dixon et al., 2015; Ou et al., 2016). Irwinda, R., et al. reported that the level of PUFAs significantly increased in PE patients (Irwinda et al., 2021; Liao et al., 2022). Furthermore, in rats model of PE, the concentration of MDA, the end product of lipid peroxidation, in the placenta has increased dramatically (Zhang et al., 2020). Similarly, the levels of MDA in plasma and placenta are significantly elevated in PE patients (Aydin et al., 2004). To summarize, these studies suggest that lipid peroxidation leading to ferroptosis could contribute to PE.

#### 4.1.2 Iron and pre-eclampsia

Iron overload is also associated with PE. Researchers have confirmed the concentration of plasma iron is higher in PE pregnancy than that in normal pregnancy (Liu et al., 2019). Yang et al., 2022a. reported the differentially expressed ferroptosis-related genes (FRGs) in early-onset PE were mainly enriched in iron-related pathways, including FTH1, FTL. Importantly, iron is abundant in trophoblasts under physiological conditions or in the context of iron deficiency (Sangkhae et al., 2020). What’s more, the expression of FPN1 of trophoblasts decreased under hypoxic conditions (Zhang et al., 2020), leading to the intracellular accumulation of Fe^2+^. Consequently, trophoblasts are vulnerable to ferroptosis. Ferrostatin-1 (Fer-1), a ferroptosis inhibitor, decreased the mortality rate of trophoblasts (Beharier et al., 2020). Similarly, the ferroptosis inhibitor improved the PE symptoms in a rat model, with the reduction of MDA (Zhang et al., 2020). Thus, reducing the concentration of Fe^2+^ might be a good way for PE treatment.

#### 4.1.3 The SLC7A11-GSH-GPX4 axis and pre-eclampsia

Disorder of antioxidant system mediates ferroptosis in PE. A microarray analysis identified that miRNA-30b-5pm, which is in charge of reducing the expression of SLC7A11, upregulated in PE placental tissues. Also, they found SLC7A11 and GPX4 were decreased in PE placental tissues *via* GSE10588 data set (Zhang et al., 2020). In accordance with other studies, the levels of SLC7A11, GSH and GPX4 declined while MDA levels were significantly increased (El-Khalik et al., 2022; D'Souza et al., 2016), indicating ferroptosis is involved in the pathogenesis of PE through the SLC7A11-GSH-GPX4 axis. A genome-wide methylome analysis found the expression of ATF3, suppressing the system Xc− by binding to the SLC7A11 promoter (Wang et al., 2020), is higher in PE placenta than the normal placenta (Ching et al., 2014). As a result, human trophoblasts are susceptible to ferroptosis by the depletion or inhibition of GPX4 (Kajiwara et al., 2022). Additionally, pannexin 1 (Panx1) and toll-like receptor 4 (TLR4), which had a negative correlation with SLC7A11, are demonstrated to induce ferroptosis in PE (El-Khalik et al., 2022). Conversely, anti-ferroptosis factors can protect trophoblasts against ferroptosis through the SLC7A11-GSH-GPX4 axis. The level of Nrf2, which is responsible for promoting transcriptions of SLC7A11 and GPX4 (Dong et al., 2020), is lower in PE rats (Ju et al., 2022). DJ-1 plays a protective role in the process of ferroptosis in PE *via* the Nrf2/GPX4 signaling pathway (Liao et al., 2022). These studies demonstrated that the SLC7A11-GSH-GPX4 axis plays a role in the pathogenesis of PE.

### 4.2 Gestational diabetes mellitus

#### 4.2.1 Lipid peroxidation and gestational diabetes mellitus

Gestational diabetes mellitus (GDM) is common during pregnancy and is increasing in prevalence globally (Sweeting et al., 2022). The incidence of GDM ranges from 6.6% to 45.3% of pregnancies (Brown and Wyckoff, 2017) and one in six live births worldwide were complicated by GDM (Atlas, 2015). GDM is associated with long-lasting complications in the short and long term, such as macrosomia (Song et al., 2022), dystocia (Crowther et al., 2022), childhood obesity in the child (Choi et al., 2022), recurrence of GDM (Giuliani et al., 2022), developing type 2 diabetes (Vounzoulaki et al., 2020) and cardiovascular disease in the mother (Christensen et al., 2022). Emerging evidence suggests ferroptosis contributes to the pathogenesis of GDM (Han et al., 2020; Gautam et al., 2021; Zhang et al., 2022c; Hu et al., 2022; Zaugg et al., 2022). The insulin sensitivity shifts depending on the requirements of pregnancy, which is an important metabolic adaptation during healthy pregnancy (Di Cianni et al., 2003). However, excessive insulin resistance in GDM promotes endogenous glucose production and the breakdown of fat stores, increasing the levels of blood glucose and free fatty acid (FFA) (Phelps et al., 1981). Indeed, glucose metabolism disorder is often accompanied by lipid metabolism disorder in GDM (Parhofer, 2015). A study indicated that women with GDM had significantly higher triglyceride (TG) concentrations (Hu et al., 2021b). *In vitro* model, the death rate of trophoblasts significantly increased after the co-treatment of high lipid (HL) and high glucose (HG). Furthermore, it was found that HL and HG can induce GDM in pregnant rats, leading to the damage of rats’ placenta (He et al., 2021). The expression of ACSL4 significantly increased in placental tissues, as well (Zheng et al., 2022). Consequently, excessive FFA of GDM may cause an increase in the level of lipid peroxidation, resulting in ferroptosis.

#### 4.2.2 Iron and gestational diabetes mellitus

Iron overload, leading to oxidative stress damage, could promote the pathogenesis of GDM (Gautam et al., 2021; Zhang et al., 2022c; Zaugg et al., 2022). As reported, both elevated plasma ferritin concentrations and iron supplementation in pregnant women having adequate iron stores are risk factors of GDM (Zhang et al., 2021). In GDM vivo model, the levels of iron deposition significantly increased (Zheng et al., 2022), inducing the production of ROS *via* the Fenton reaction. As a result, oxidative damage leads to the injury and ferroptosis of pancreatic β–cell in GDM (Gautam et al., 2021; Du et al., 2022). The SLC7A11-GSH-GPX4 axis also contributes to GDM. The serum lipid peroxidation was higher, while the serum GPX4 concentration was lower in GDM women (Mauri et al., 2021). In summary, mounting evidence may suggest that excessive iron, and reduced GPX4 levels, two hallmarks of ferroptosis, are associated with GDM. However, experiments testing this hypothesis are still lacking.

### 4.3 Intrahepatic cholestasis of pregnancy

Intrahepatic cholestasis of pregnancy (ICP) is a complication, most occurs in the third trimester, in 0.3%–15% of pregnancies in various populations (Wikström Shemer et al., 2013). It is characterized by pruritus, elevated serum bile acid levels and liver transaminases, leading to meconium-stained amniotic fluid, fetal distress, preterm birth, and stillbirth (Wikström Shemer et al., 2013). There is increasing evidence that oxidative stress induced by bile acids leads to the pathogenesis of ICP (Sanhal et al., 2018). ICP patients had significantly lower levels of Se and GPX4 than normal pregnancies (Reyes et al., 2000; Hu et al., 2015). Moreover, patients with ICP had significantly higher level of MDA (Zhu et al., 2019). Analysis of differentially expressed ferroptosis-related genes in ICP and healthy pregnant showed EGFR, mediating ferroptosis, was higher upregulated in human placenta (Fang and Fang, 2022). Modification of oxidative stress caused by ferroptosis might be a treatment target for ICP. Further research, particularly *in vivo* and *in vitro* experiments, is needed to characterize the association between ferroptosis and ICP.

### 4.4 Other pregnancy-related disease

Excessive ferroptosis occurred in spontaneous abortion rat model with low levels of GSH, GPX4 and increased levels of TFR1, ACSL4 and MDA (Meihe et al., 2021). Some evidence also indicated spontaneous preterm birth is related to ferroptosis (Beharier et al., 2020). But few studies reported that the exact mechanism of ferroptosis and spontaneous abortion and spontaneous preterm birth are still unclear.

## 5 Potential medicines for ferroptosis in pregnancy related diseases

Trophoblast ferroptosis may provide a useful therapeutic target for pregnancy-related diseases. Quercetin, as an antioxidant, can significantly promote trophoblast invasion during early pregnancy *via* significantly increasing GSH levels (Ebegboni et al., 2019). Additionally, quercetin has positive effects on pre-eclampsia rats induced by L-NAME (Yang et al., 2019a; Yang et al., 2022b). Iron chelators, deferoxamine and ferrostatin-1, were indicated to decrease the concentration of placenta MDA in the PE rat mode, thereby blocking trophoblast ferroptosis (Beharier et al., 2020; Zhang et al., 2020). Similarly, vitamin E plays a role in the preventing PE by mitigating lipid peroxidation in placenta (Raijmakers et al., 2004). Thiazolidinediones, inhibiting ACSL4 against ferroptosis, is also oral antidiabetic drug by sensitizing tissue to the effects of insulin (Pollex and Hutson, 2011). A recently published case series demonstrate thiazolidinediones is safe during pregnancy (Haddad et al., 2008), indicating its potential therapeutic role for GDM. Trophoblasts ferroptosis may contribute to ICP, while the low concentration of Se is related to the pathogenesis of ICP (Reyes et al., 2000). Thus, Se, upregulating the expression of GPX4, can protect placental trophoblasts against oxidative stress, particularly ICP (Habibi et al., 2021). CoQ10 is significantly decreased in patients with ICP (Martinefski et al., 2014). Furthermore, CoQ10 supplementation improves estradiol-induced cholestasis in rats. CoQ10 supplementation is very well tolerated and has no clinically relevant toxic side effects in humans (Hidaka et al., 2008; Martinefski et al., 2020). Therefore, it would be an alternative therapy for women with ICP. Lack of 1,25(OH)_2_D_3_ is related to PRDs, which may result from ferroptosis, such as spontaneous abortion, GDM and PE (Bespalova et al., 2019; Li et al., 2019; de Souza and Pisani, 2020). Vitamin D elevated the level of GSH, GPX4 and reduced MDA through activation of the Nrf2/HO-1 pathway to suppresses ferroptosis. Therefore, vitamin D supplementation may be a strategy to improve PRDs. Previous studies reported astaxanthin significantly reduced the content of MDA in preeclamptic rats and trophoblast cell line (Xuan et al., 2014; Xuan et al., 2016; Fu et al., 2021). Certainly, drug efficacy and safety are quite important for pregnant woman and fetus. Further research may shed light on potential targeting drugs for ferroptosis in PRDs.

## 6 Conclusions and perspectives

Ferroptosis is a form of regulated cell death involving lipid metabolism, iron metabolism and antioxidant system, regulated by multiple genes and signaling pathways. PRDs are mainly associated with placenta dysfunction due to trophoblasts injury and death. Recently, an increasing number of experimental studies are exploring role of ferroptosis in PRDs in order to provide new potential therapeutic drugs and therapeutic targets for it. However, there are numerous problems that have not been elucidated on the association between ferroptosis and pregnancy related diseases. Firstly, the exact molecular mechanism of transplacental iron transport is not clear, though much work has been done on it. Secondly, ferroptosis is a form of cell death that is associated with lots of signaling pathways, like hypoxia signaling (Zou et al., 2019), AMP-activated protein kinase signaling (Li et al., 2020), E-cadherin-NF2-Hippo-YAP pathway (Yang et al., 2019b), and NRF2-KEAP1 pathway (Anandhan et al., 2020). However, the regulation of ferroptosis in placenta also remains a pressing challenge. Finally, we still do not know whether ferroptosis of trophoblasts leads to PRDs, or it is the execution pathway of PRDs. Therefore, extensive investigation is needed to explore it.

## Abbreviations

AA-CoA, arachidonic acid-CoA; AA-PE, arachidonic acid-phosphatidylethanolamine; AdA-CoA, adrenic acid-CoA; AdA-PE, adrenic acid-phosphatidylethanolamine; ACSL4, acyl-CoA synthetase longchain family member 4; AIFM2, apoptosis-inducing factor 2; CPX, ciclopirox; CHMP5, charged multivesicular body protein 5; CHMP6, charged multivesicular body protein 6; DFO, deferoxamine; DMT1, divalent metal transporter 1; ESCRT-III, endosomal sorting complexes required for transport-III; FTH1, ferritin heavy chain 1; FTL, ferritin light chain; GCLC, glutamate cysteine ligase, catalytic; GPX4, glutathione peroxidase 4; GSH, glutathione; GSS, glutathione synthetase; HMG-CoA, β-Hydroxy β-methylglutaryl-CoA; HMGCR,3-hydroxy-3-methylglutaryl-CoA reductase; LDs, Lipid droplets; LOX, lipoxygenase; LPCAT3, lysophosphatidylcholine acyltransferase 3; NCOA4, nuclear receptor coactivator 4; PL, phospholipid; RSL3, ras-selective lethal 3; ROS, reactive oxygen species; Se, selenium; STEAP3, six-transmembrane epithelial antigen of prostate 3; TF, transferrin; TfR1, transferrin receptor-1.
